# 3-Bromo-4,5-dihydroxybenzaldehyde Enhances the Level of Reduced Glutathione via the Nrf2-Mediated Pathway in Human Keratinocytes

**DOI:** 10.3390/md15090291

**Published:** 2017-09-18

**Authors:** Ki Cheon Kim, Yu Jae Hyun, Susara Ruwan Kumara Madduma Hewage, Mei Jing Piao, Kyoung Ah Kang, Hee Kyoung Kang, Young Sang Koh, Mee Jung Ahn, Jin Won Hyun

**Affiliations:** 1 Jeju National University School of Medicine and Jeju Research Center for Natural Medicine, Jeju 63243, Korea; svv771@hotmail.com (K.C.K.); yujae_1113@naver.com (Y.J.H.); susaramh@gmail.com (S.R.K.M.H.); meijing0219@hotmail.com (M.J.P.); legna07@naver.com (K.A.K.); pharmkhk@jejunu.ac.kr (H.K.K.); yskoh7@jejunu.ac.kr (Y.S.K.); 2 Laboratory of Veterinary Anatomy, College of Veterinary Medicine, Jeju National University, Jeju 63243, Korea; healthy@jejunu.ac.kr

**Keywords:** 3-bromo-4,5-dihydroxybenzaldehyde, glutathione, oxidative stress, glutathione synthetase, NF-E2 related factor 2

## Abstract

A natural bromophenol found in seaweeds, 3-bromo-4,5-dihydroxybenzaldehyde (BDB), has been shown to possess antioxidant effects. This study aimed to investigate the mechanism by which BDB protects skin cells subjected to oxidative stress. The effect of BDB on the protein and mRNA levels of glutathione-related enzymes and the cell survival of human keratinocytes (HaCaT cells) was investigated. BDB treatment increased the protein and mRNA levels of glutathione synthesizing enzymes and enhanced the production of reduced glutathione in HaCaT cells. Furthermore, BDB activated NF-E2-related factor 2 (Nrf2) and promoted its localization into the nucleus by phosphorylating its up-stream signaling proteins, extracellular signal–regulated kinase and protein kinase B. Thus, BDB increased the production of reduced glutathione and established cellular protection against oxidative stress via an Nrf2-mediated pathway.

## 1. Introduction

The oxidative stress induced by reactive oxygen species (ROS), including oxidants such as hydrogen peroxide and free radicals such as hydroxyl radicals and superoxide anions, is the prime cause of skin aging [1,2]. Hence, it is important for cells to control the ROS levels and prevent their accumulation. Cells are known to follow two pathways to cope with increasing ROS levels: the early responsive mechanism and the delayed responsive mechanism. The early responsive mechanism rapidly removes ROS and free radicals through chemical reactions whenever they are generated [3]. The delayed responsive mechanism involves the expression of genes encoding antioxidant enzymes and proteins to reduce the generation of noxious substances, as well as the expression of genes encoding substances that regulate cellular activities such as signal transduction, proliferation, and immunologic defense reactions [4]. A number of various factors have been found to be associated with cellular responses to oxidative stress. NF-E2-related factor 2 (Nrf2), protein kinase B, and NF-kappa B activate cell survival, whereas c-Jun *N*-terminal kinase (JNK), p38 kinase, and p53 induce cell cycle arrest and apoptosis [5]. Nrf2, a pivotal transcription factor of phase II antioxidant enzymes, is referred to as the central signaling switch that induces antioxidant enzymes such as glutamate-cysteine ligase catalytic subunit (GCLC) and glutathione synthetase (GSS) [6]. During oxidative stress, Nrf2 binds to the antioxidant response elements (ARE) in the promoters of GCLC and GSS to initiate the transcription of these genes [7,8].

GCLC and GSS are enzymes for the synthesis of reduced glutathione (GSH), which is an important antioxidant that maintains the redox status. Apart from scavenging free radicals, GSH is able to ensure the redox-sensitive active sites of many enzymes via converting the reduced form from the oxidized form [9,10]. Hence, maintaining the optimum balance between GSH and oxidized glutathione (GSSG) is important for cellular defense against oxidative stress [10].

Natural compounds from seaweeds are known to enhance the cellular defense mechanisms against oxidative stress. In particular, 3-bromo-4,5-dihydroxybenzaldehyde (BDB), a natural antioxidant mainly found in red algae, has been shown to exhibit antiviral activity against the hematopoietic necrosis virus and the infectious pancreatic necrosis virus [11]. We had previously shown that BDB could also protect human keratinocytes from apoptosis by diminishing DNA damage, lipid peroxidation, and protein carbonylation after UVB exposure [12]. In this study, we focused on the ability of BDB to enhance GSH by increasing GCLC and GSS expression through the Akt/Nrf2 pathway.

## 2. Results

### 2.1. BDB Induces the Expression of GSH-Synthesizing Enzymes

**Figure 1:**
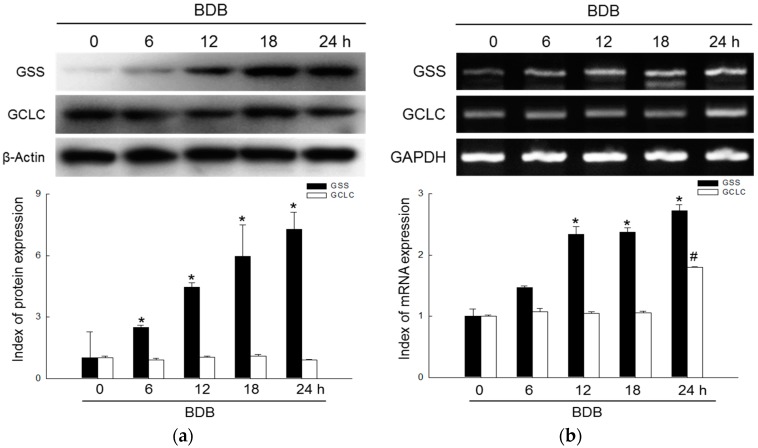
Effects of BDB on expression of GSH-synthesizing enzymes, GSS and GCLC, in HaCaT cells. (**a**) Protein levels of GSS and GCLC detected using Western blot assay. * indicates significant differences from control (*p* < 0.05); (**b**) mRNA levels of GSS and GCLC detected using RT-PCR. *, # indicates significant differences from control of GSS and GCLC, respectively (*p* < 0.05).

The expression levels of GSS and GCLC proteins in HaCaT cells were examined at 0, 6, 12, 18, 24 h with BDB treatment. The results of Western blot assays revealed a significant elevation of GSS after 12 h of incubation with 10 μM BDB (Figure 1a). These results were confirmed using RT-PCR, which revealed that GSS mRNA levels were notably increased after 6 h of incubation with BDB (Figure 1b). GCLC did not show similar variations as GSS in the Western blot assay and RT-PCR data was also not similar to GSS, but in incubation 24 h after BDB treatment, there was a significant increase in the GCLC mRNA levels (Figure 1a,b).

### 2.2. BDB Shields HaCaT Cells from UVB through GSS

**Figure 2:**
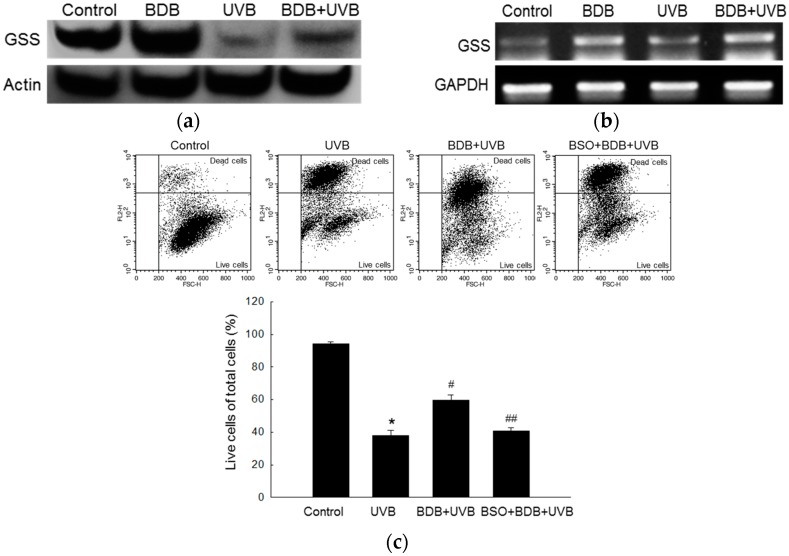
Effects of BDB on GSS in UVB-exposed HaCaT cells. (**a**) Western blot assay and (**b**) RT-PCR analysis were performed to detect protein and mRNA levels of GSS, respectively; (**c**) The populations of live cells and dead cells were detected using flow cytometry after PI staining, and dot plots were generated. *, #, ## indicate significant differences from control, UVB, and BDB + UVB groups, respectively (*p* < 0.05).

We next investigated variations in GSS levels in BDB-pretreated HaCaT cells after UVB exposure. GSS is essential for the first step of GSH biosynthesis, and so, fluctuations in GSH levels were assayed using Western blot and RT-PCR. BDB treatment caused an obvious increase in the protein and mRNA levels of GSS in human keratinocytes, compared to the controls (Figure 2a,b), while UVB exposure caused a decrease in GSS levels. However, it is to be noted that BDB pretreatment recovered the UVB-induced GSS loss. These results indicated that BDB attenuated a UVB-induced decrease of GSS through GSS induction. In addition, UVB exposure caused 63% cell death, compared to the controls, as measured by propidium iodide (PI) staining and flow cytometry analysis. Cells treated with BDB alone showed 41% cell death after UVB exposure, whereas cells treated with both BDB and L-buthionine-sulfoximine (BSO) (100 μM), which is GSS enzyme activity inhibitor [13], showed 59% cell death after UVB irradiation (Figure 2c).

### 2.3. BDB Induces Activation of Nrf2 and Enhances Binding of Nrf2 to AREs in the Promoter of the GSS Gene

**Figure 3:**
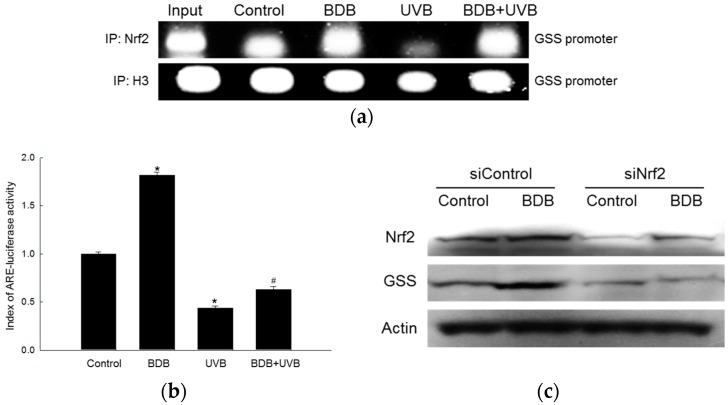
Effects of BDB on the binding of Nrf2 to ARE, Nrf2 transcriptional activity in UVB-exposed HaCaT cells, and GSS expression in siNrf2-transfected HaCaT cells. (**a**) Nuclear extracts were prepared and ChIP assay was performed to measure the binding of Nrf2 to ARE in the promoter of the GSS gene. Histone H3 was used the internal control; (**b**) Transcriptional activity of Nrf2 was measured using the ARE-luciferase reporter assay. *, # indicate significant differences from control and UVB groups, respectively (*p* < 0.05); (**c**) GSS expression was detected using Western blot analysis in control siRNA (siControl)- and Nrf2 siRNA (siNrf2)-transfected cells.

After showing that BDB induced GSS expression and protected HaCaT cells from UVB-induced oxidative damage, we focused on transcription factor Nrf2, known as the central signaling switch that modulates the activation of phase II antioxidant enzymes, including GSS [6]. The binding of Nrf2 to the AREs in the promoter of the GSS gene was markedly increased in BDB-treated cells; furthermore, BDB-pretreatment significantly increased the binding of Nrf2 to AREs in the promoters of the GSS gene after UVB exposure, compared to the UVB controls, as measured using the chromatin immune-precipitation (ChIP) method (Figure 3a). In addition, when a construct containing an ARE sequence (with the Nrf2-binding site) linked to a luciferase reporter gene was transfected into the HaCaT cells, BDB treatment increased the transcriptional activity of Nrf2 (Figure 3b) compared to the controls. Furthermore, BDB pretreatment prior to UVB exposure attenuated the UVB-induced loss of Nrf2 transcriptional activity. To confirm the elevation of Nrf2 activation by BDB, a Western blot assay was performed on Nrf2-silenced HaCaT cells (Figure 3c). The GSS levels were not elevated in the siNrf2 group compared to the siControl group.

### 2.4. BDB Induces Activation of Cell Survival Signal

**Figure 4:**
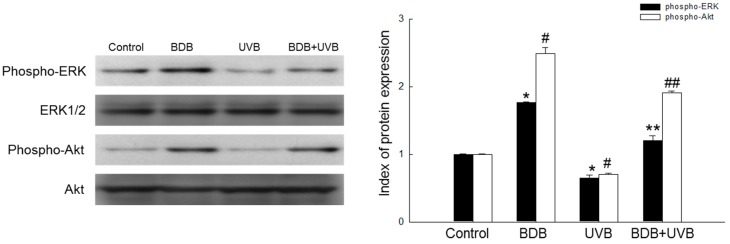
Effects of BDB on ERK1/2 and Akt proteins in UVB-exposed HaCaT cells. Western blot analysis was used to detect phospho-ERK and phospho-Akt proteins. ERK1/2 and Akt were used as loading controls. *, # indicate significant differences from control of phospho-ERK and phospho-Akt, respectively (*p* < 0.05). **, ## indicate significant differences from UVB group of phospho-ERK and phospho-Akt, respectively (*p* < 0.05).

Several kinases such as phosphoinositol 3-kinase (PI3K)/protein kinase B (Akt) and mitogen-activated protein kinases (MAPKs/ERK1/2) have been reported to be involved in the activation of Nrf2 and the up-regulation of several antioxidant enzyme genes in different cells [14]. The phosphorylation of serine and threonine residues of Nrf2 by these kinases is known to facilitate the nuclear translocation of Nrf2 and its subsequent binding to the coactivator, CBP/p300 [15]. We assessed the up-stream signaling pathway involved in the BDB-mediated activation of Nrf2 and the induction of GSS to analyze the activation of Akt and ERK1/2. A marked increase in phosphorylated ERK (phospho-ERK) and phosphorylated Akt (phospho-Akt) was observed in BDB-treated HaCaT cells. Furthermore, compared to the UVB-exposed group, phospho-ERK and phospho-Akt were increased in the BDB-pretreated group (Figure 4).

### 2.5. BDB Promotes the Synthesis of Reduced GSH Catalyzed by Glutathione Synthesizing Enzymes

**Figure 5:**
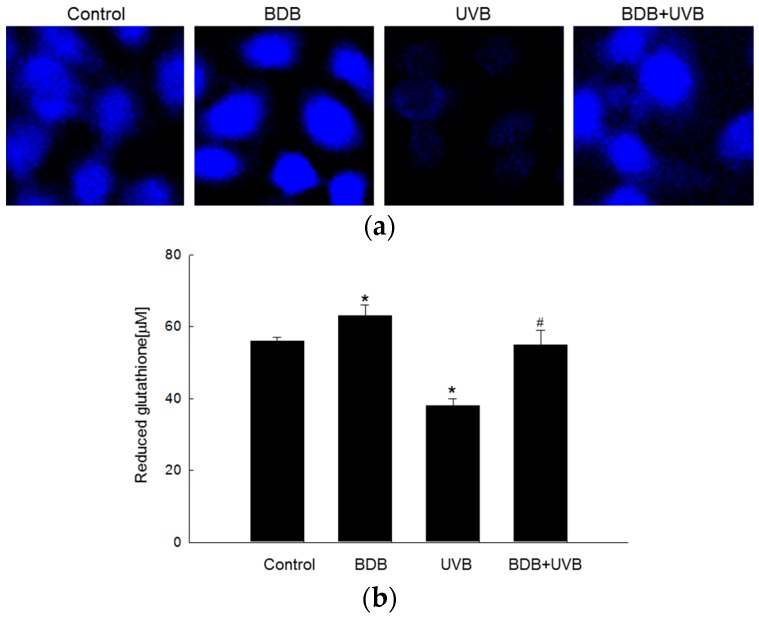
Effects of BDB on GSH levels in UVB-exposed HaCaT cells. GSH level was measured (**a**) by confocal microscopy after CMAC staining and (**b**) by a GSH detection kit in the indicated groups. *, # indicate significant differences from control and UVB groups, respectively (*p* < 0.05).

Reduced glutathione, which is the most prevalent low-molecular-weight antioxidant in cells, protects cellular components from oxidative injuries, either by reacting directly with oxidants or by acting as a substrate for radical scavenging by glutathione peroxidase [16]. GSH levels were analyzed using confocal microscopy after staining with a GSH-specific probe, 7-amino-4-chloromethylcoumarin (CMAC). The fluorescence intensity of CMAC was proportional to the level of GSH, which was higher in the BDB-treated cells than the controls. The level of GSH was significantly reduced after UVB exposure and restored in BDB-pretreated cells (Figure 5a). The confocal microscopic data were consistent with the data from a GSH detection kit (Figure 5b).

## 3. Discussion

Oxidative stress is known to change the composition and structure of cellular compounds such as DNA, lipids, and proteins by generating ROS [17,18], causing mutations, interruption of enzymatic activities, and degradation of cellular structures, ultimately leading to apoptosis [19]. Fortunately, cells have evolved antioxidant defense systems to maintain ROS concentrations at desirable levels [13]. GSH, a tripeptide antioxidant composed of glycine, cysteine, and glutamic acid, is known to attenuate ROS-induced damage to cellular components [20]. Glutathione acts as an electron donor to reduce disulfide bonds formed in cytoplasmic proteins into cysteine. During oxidations, GSH is converted to its oxidized form, glutathione disulfide (GSSG); glutathione can be converted from GSSG to reduced glutathione by glutathione reductase, using an electron donor, NADPH [21]. GSS and GCLC are key enzymes that maintain the GSH redox status [22]. Therefore, these two enzymes are important targets for enhancing the cellular defense against oxidative stress using pharmacological agents.

A bromophenol isolated from red algae including BDB is known to possess anticancer and antioxidant activities [23]. A recent study demonstrated that the bromophenol compound also has anti-angiogenesis capabilities [24]. In this study, we examined the protective effects of BDB in human keratinocytes against oxidative stress through Nrf2-mediated GSH synthesis. Our previous studies had shown that 10 μM of BDB could efficiently scavenge ROS generated by UVB and shield HaCaT cells against UVB-induced apoptosis by attenuating DNA fragmentation, lipid peroxidation, and protein carbonylation [12]. Therefore, in this study, we chose 10 μM as the optimum dose of BDB. Our results demonstrated that 10 μM of BDB could effectively increase the protein and mRNA levels of GSS. Interestingly we did not observe a similar trend for the levels of GCLC. GSS is a key enzyme in the first step of GSH synthesis, and contributes to the survival of cells under oxidative stress. We confirmed that BDB exerts its protective effect by stimulating GSS expression in HaCaT cells. Flow cytometry, followed by PI staining, indicated that BDB pretreatment reduced UVB-induced cell death. Keratinocytes treated with BSO, a GSS inhibitor, and BDB did not show a significant elevation in survival after UVB exposure. Cells treated with BDB also showed a notable increase in the protein and mRNA levels of GSS after UVB exposure, confirming that BDB protected HaCaT cells from oxidative stress (UVB) by elevating the level of GSS (Figure 2).

Nrf2 plays an important role in the up-regulation of antioxidant and detoxifying enzymes. Recent studies have shown that failure of the Nrf2 function promotes carcinogen-induced DNA damage and tumor formation [7,25]. Therefore, Nrf2 is an important molecular target for cancer prevention [26]. During oxidative stress, the cysteine residues of Keap1 are oxidized, causing a conformational change in the Keap1-Nrf2 complex. This conformational change prevents Keap1 from ubiquitinating Nrf2, and stimulates the translocation of Nrf2 into the nucleus, where it binds to AREs, initiating the transcription of downstream target genes such as the GSS gene [27,28]. The results of ChIP and luciferase reporter gene assays (Figure 3a,b) showed that BDB enhanced the binding of Nrf2 to the ARE in the GSS gene promoter, in both UVB and non-UVB groups. To further confirm the increase in GSS levels by BDB through Nrf2 activation, HaCaT cells were transfected with siNrf2. As expected, the Nrf2-silenced groups did not exhibit increased levels of GSS, compared to the siControl, as measured using a Western blot assay (Figure 3c). To study the signaling pathway for the BDB-mediated activation of Nrf2, we analyzed the phosphorylation of ERK1/2 and Akt. The protein levels of phospho-ERK and phospho-Akt were reduced in UVB-exposed cells, but were restored upon BDB pretreatment (Figure 4). ERK1/2 and Akt signaling pathways are essential for the BDB-induced Nrf2 activation and subsequent expression of GSS. Taken together, these results showed that BDB protected HaCaT cells against oxidative stress by activating the transcription of GSS by inducing Nrf2 translocation to the nucleus.

Besides HaCaT cells, GSH is an essential antioxidant in many cells, including neurons. GSH depletion could significantly affect the survival of dopamine neurons, especially under oxidative stress, and loss of GSH may be an early event in Parkinson’s disease [29]. Several studies have examined the response of brain cells to GSH depletion. BSO treatment causes the depletion of GSH in the brain and damages the mitochondria [30], suggesting that GSH-dependent reactions are important for the removal of H_2_O_2_ from the mitochondria. The previous study demonstrated that 70 mJ/cm^2^ as well as 30 mJ/cm^2^ UVB exposure in HaCaT cells decreased the GSH level [31]. Therefore, further study on the GSH-enhancing effect of BDB at different UVB exposures is required. We also investigated the variations in GSH levels in HaCaT cells, in response to BDB treatment. Confocal microscopy, followed by CMAC staining, revealed that BDB increased GSH expression, as well as significantly attenuated UVB-induced loss of GSH (Figure 5). These results were confirmed by quantifying the GSH levels using a GSH detection kit.

**Figure 6:**
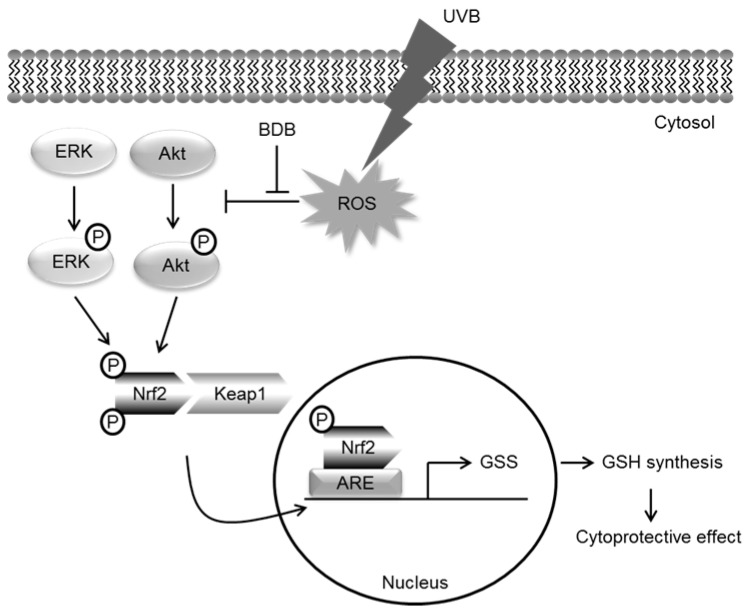
Schematic hypothesis of cytoprotective effect of BDB against UVB-exposed HaCaT cells via Nrf2-GSH signaling pathway.

Therefore, BDB clearly exerted cellular protection by stimulating the synthesis of GSH through Nrf2 activation. In summary, these results confirmed that the protective mechanism of BDB against UVB-induced oxidative stress involved establishing GSH balance through the Nrf2-mediated pathway (Figure 6).

## 4. Materials and Methods

### 4.1. Cell Culture and UVB Irradiation

Human keratinocytes, HaCaT cells, were cultured in an RPMI 1640 medium with 10% fetal bovine serum and exposed to 30 mJ/cm^2^ of UVB (CL-1000M UV Crosslinker, Upland, CA, USA).

### 4.2. Reverse Transcription-PCR (RT-PCR)

PCR was performed under the following conditions: 26 cycles at 94 °C for 30 s, 63 °C for 45 s, and 72 °C for 1 min. The primers used were: human GCLC, forward 5′-AACCAAGCGCCATGCCGACC-3′ and reverse 5′-CCTCCTTCCGGCGTTTTCGC-3′; human GSS, forward 5′-GCCCCATTCACGCTCTTCCCC-3′ and reverse 5′-ATGCCCGGCCTGCTTAGCTC-3′; and human GAPDH, forward 5′-TCAAGTGGGGCGATGCTGGC-3′ and reverse 5′-TGCCAGCCCCAGCGTCAAAG-3′. The PCR products were subjected to electrophoresis and analyzed using image analysis software.

### 4.3. Western Blot Analysis

Harvested cells were lysed by incubating on ice and centrifuged. The supernatant was collected and 30 μg of protein was electrophoresed on 12% SDS-polyacrylamide gels. The separated proteins were transferred onto nitrocellulose membranes and incubated with the primary antibodies (dilution by 1% BSA solution at 1:1000), followed by horseradish peroxidase secondary antibody conjugates (dilution by 1% BSA solution at 1:10,000). The protein bands were detected using a Western blotting detection kit. Antibodies against the following proteins were used in this study: Nrf2 (Cell Signaling Technology, Beverly, MA, USA), Akt (Cell Signaling Technology), phospho-Akt (Cell Signaling Technology), ERK (Santa Cruz Biotechnology, Santa Cruz, CA, USA), phospho-ERK (Santa Cruz Biotechnology), GCLC (Santa Cruz Biotechnology), GSS (Santa Cruz Biotechnology), and β-actin (Sigma-Aldrich Chemical Company, St. Louis, MO, USA).

### 4.4. Chromatin Immune-Precipitation (ChIP) Analysis

The cells were first processed using enzymatic chromatin IP kit (Cell signaling technology), according to the manufacturer’s instructions. Nrf2 antibody (Cell signaling Technology) was used for ChIP analysis. The ChIP procedure was performed by PCR using the following human GSS promoter-specific primers: forward 5′-CTGGGAATAACCAGACACCTA-3′ and reverse 5′-CAGGTTCAAGCAATTCTCCTG-3′. The PCR cycle conditions were as follows: initial denaturation at 95 °C for 5 min; 40 cycles of 95 °C for 30 s, 60 °C for 30 s, and 72 °C for 30 s; and a final extension at 72 °C for 7 min. The amplified products were resolved using electrophoresis and analyzed using image analysis software.

### 4.5. Determination of the Cell Viability

HaCaT cells were treated with 10 μM BDB (Matrix Scientific, Columbia, SC, USA) for 1 h and then treated BSO (Cayman Chemical, Ann Arbor, MI, USA) and exposed to UVB. After 24 h, the cells were stained using propidium iodide. The fluorescence of the cells was measured using a flow cytometer (FACSCalibur flow cytometer, Becton Dickinson, Mountain View, CA, USA).

### 4.6. Detection of GSH

Cell Tracker™ Blue CMAC (Molecular Probes, Eugene, OR, USA) was used to detect the GSH level. GSH levels were measured using a confocal microscope after staining with CMAC. In addition, the GSH concentration was detected using the BIOXYTECH GSH-400 assay kit (Foster City, CA, USA).

### 4.7. Transient Transfection of Small Interfering RNA (siRNA)

The siRNA constructs used were a mismatched siRNA control (siControl, Santa Cruz Biotechnology) and a siRNA against Nrf2 (siNrf2, Santa Cruz Biotechnology). The cells were transfected with 10–50 nM siRNA using Lipofectamine™ 2000 (Invitrogen, Carlsbad, CA, USA), according to the manufacturer’s instruction. At 24 h after transfection, the cells were treated with 10 μM of BDB for 24 h and subjected to a western blot analysis.

### 4.8. Luciferase Activity

HaCaT cells were transiently transfected with 0.5 μg of the Nrf2-binding site-ARE luciferase plasmid vector using Lipofectamine™ 2000 (Invitrogen Corporation). The cells were then co-transfected with 0.02 μg of pRL-TK *Renilla reniformis* luciferase, which served as a normalizing control. Luciferase assays were performed using the dual luciferase assay system (Promega, Madison, WI, USA). The ARE-luciferase reporter gene was kindly provided by Professor Young-Joon Surh of Seoul National University (Seoul, Korea).

### 4.9. Statistical Analysis

All measurements were performed in triplicate and all values were expressed as the mean ± standard error. The results were subjected to an analysis of variance, followed by Tukey’s test to analyze the differences between means. In each case, *p* < 0.05 was considered statistically significant.
